# Gold nanoparticles - the theranostic challenge for PPPM: nanocardiology application

**DOI:** 10.1186/1878-5085-4-18

**Published:** 2013-06-24

**Authors:** Mykola Ya Spivak, Rostyslav V Bubnov, Ilya M Yemets, Liudmyla M Lazarenko, Natalia O Tymoshok, Zoia R Ulberg

**Affiliations:** 1 Zabolotny Institute of Microbiology and Virology, National Academy of Sciences of Ukraine, Zabolotny Str., 154, Kyiv 03680, Ukraine; 2 LCL “DIAPROF”, Svitlycky Str., 35, Kyiv 04123, Ukraine; 3 Clinical Hospital “Pheophania” of State Affairs Department, Zabolotny Str., 21, Kyiv 03680, Ukraine; 4 Scientific-Practical Centre of Pediatric Cardiology and Cardiac Health of Ukraine, Chornovil Str., 28/1, Kyiv 01135, Ukraine; 5 Ovcharenko Institute of Biocolloidal Chemistry, National Academy of Sciences of Ukraine, Acad. Vernadsky Blvd, 42, Kyiv 03142, Ukraine

**Keywords:** Predictive, Preventive and Personalised Medicine, Nanomedicine, Gold Nanoparticles, Drug Delivery, Sonoporation, Theranostics

## Abstract

The article overviews the potential biomedical applications of nanoscale gold particles for predictive, preventive and personalised nanomedicine in cardiology. The review demonstrates the wide opportunities for gold nanoparticles due to their unique biological properties. The use of gold nanoparticles in cardiology is promising to develop fundamentally new methods of diagnosis and treatment. The nanotheranostics in cardiovascular diseases allows the non-invasive imaging associated with simultaneous therapeutic intervention and predicting treatment outcomes. Imaging may reflect the effectiveness of treatment and has become a fundamental optimisation setting for therapeutic protocol. Combining the application of biomolecular and cellular therapies with nanotechnologies foresees the development of complex integrated nanodevices. Nanocardiology may challenge existing healthcare system and economic benefits as cardiovascular diseases are the leading cause of morbidity and mortality at present.

## Review

### Introduction

#### Predictive, preventive and personalised cardiovascular nanomedicine

Heart diseases are one of the main causes of death worldwide; heart failure is associated with a significantly reduced physical and mental health, resulting in a decreased quality of life [1,2]. Although many patients with cardiovascular diseases survive for many years, progressive disease is associated with an overall annual mortality rate of 10% [3]; heart failure is the leading cause of hospitalisation in people older than 65 years [4]. One of the outstanding achievements at the end of the last century are the studies on properties of biological and synthetic materials in nanometre. The rapid development of nanoscience has caused the formation of fundamentally new directions for biotechnology research nano-objects, which are characterised by peculiar, often unexpected properties that are different from the properties of both macro- and microscale particles.

Advances in nanoscience, nanotechnology and nanomedicine lead to the construction of new materials and devices for various scientific and therapeutic purposes, which are applicable in molecular diagnostics, nanodiagnostics and improvements in the discovery, design and delivery of drugs, including nanopharmaceuticals, promising to enhance the ability of clinicians to address some of the serious challenges responsible for cardiovascular mortality, morbidity and numerous societal consequences [5]. Nanotechnology and nanomaterials have to find a wide application in cardiology and vascular therapy in the treatment of patients with venous and arterial thrombosis, the manufacture of intravascular and intracardiac implants, the creation of vascular tissue, etc. [1,5].

In developing the paradigm of *predictive*, *preventive and personalised medicine*, a crucial point is to diagnose, observe the process of tissue transformation treatment and analyse early parameters (biomarkers) to assess/predict the treatment outcome, driven by a decision making process [6]. One of the goals of personalised medicine is highly specific and sensitive drug targeting, i.e. giving the patients the right drug for their disease at the right dose and the right time.

Nanobiotechnology will facilitate the integration of diagnostics with therapeutics for personalised medicine, i.e. prescribing specific therapeutics best suited for an individual. Many of the developments have already started, and within a decade, a definite impact will be felt in the practice of medicine [7].

It was reported in the *EPMA Journal*[8] that biomarkers can also be categorised as pharmacodynamic, prognostic or predictive. Pharmacodynamic biomarkers indicate the outcome of the interaction between a drug and a target, including both therapeutic and adverse effects [9]. The study on drug delivery by nanoparticles is a highly perspective direction of personalised medicine in the future [10].

Optoacoustic phenomena and other still hidden properties of nanomaterials open new views on personalised and predictive approach and could be the basis of creating pharmacodynamic biomarkers ideal for use in diagnostics and prognostics, being able to suggest the likely outcome of a disease irrespective of treatment. Nanobiotechnology forms the basis of many new devices being developed for medicine and surgery such as nanorobots [7]. The *aim* of the article is to give an overview of the potential biomedical applications of nanoscale gold particles in cardiology for predictive, preventive and personalised nanomedicine.

### Gold nanoparticles: the types and physical, chemical and biological properties

Nanotechnology has a very broad definition based on scale, and nanomedicines are likewise based not only on the type of medicine or their function but also on the nanosize range. While most nanotechnology is expected to have an upper size limit of 100 nm, in the drug delivery field, this is more generally accepted as medicines in the size range from a few nanometres to 1,000 nm in diameter.

*Nanoparticles* (NPs), thanks to their structural features, have unique physical, chemical and biological properties and functional activity [7,11]. This phenomenon to a large extent depends on the nanoparticle size and shape, which are connected with surface area and quantum effects. Reducing the size of the nanoparticles leads to the fact that, compared with internal content, a significantly greater proportion of atoms (the components of the nanoparticles) is on the surface. Thus, it is reported that for particles with a size of 30 nm, about 5% of the atoms is on their surface, and for those with sizes of 10 and 3 nm, about 20% and 50% of the atoms is on their surface, respectively [11-14]. Thus, nanometre-size particles have a much larger surface area per unit mass than those of larger size. This presents unique properties of nanomaterials and leads to the search of new and more advanced methods of controlled synthesis and the establishment of mechanisms of nanoparticle property dependence on size and shape.

Among the nanocarriers, colloidal gold particles are a lead candidate in the field of nanotechnology, thanks to its chemical, physical, pharmacological and optical properties, and have broad prospects for introduction in medical and veterinary practice. Their unique physical and chemical properties, such as inertia, stability, biocompatibility, low level of cytotoxicity and others, cause significant medico-biological potential of gold nanoparticles and determine the prospects of their wide use as vectors for targeted delivery of drugs, for the creation of biosensors to detect toxins, as well as contrast agents which are more effective than the standard drugs base on iodine-derived compounds [2-8]. In particular, promising is the creation of nanoconstructions based on gold nanoparticles (AuNPs) and cardiotropic drug delivery to increase the clinical efficacy of treatment of patients with heart failure because of their unique biological properties [11,12].

All *plasmonic* (noble metal) nanoparticles distinguish themselves from other nanoplatforms such as semiconductor quantum dots and magnetic and polymeric nanoparticle by their unique *surface plasmon resonance*[13-15]. Nanogold (gold nanoparticle, colloidal gold) has been actively investigated in a wide variety of biomedical applications due to its biocompatibility and ease of conjugation to biomolecules [11,16] and thus offers multiple modalities for biological and medical applications [17].

Today we know a few gold nanoparticles, the characteristics of which are due to their shape and size: spheres, rods, hexagonals, etc. [11]. Thanks to their unique physical properties, combined nanoparticles, in which gold is used for the synthesis of the kernel or to cover the surface of nanoparticles (thecal structure), have been intensively investigated [11]. The non-cytotoxicity, non-immunogenicity and biocompatibility of many AuNPs make us relatively optimistic concerning their future essential applications in nanomedicine [11,18,19].

#### Preparation of AuNPs

As is known so far, the synthesis of gold nanoparticles can be conditionally divided into two groups: dispersion and condensation. Dispersion methods of obtaining gold nanoparticles are based on the destruction of the crystal lattice of the gold metal under the action of high-voltage electric current. Condensation methods are more common than dispersion and are distinguished as physical and chemical. The formation of nanoparticles in these methods is carried out through a number of intermediate states that give rise to the nuclei of the new phase, with spontaneous growth and orientation of the physical surface of the phase.

The *condensation methods* for the restoration of gold halides (for example, HAuCl4) are mostly used with chemical restoration and/or ultrasonic, ultraviolet radiation, pulsed or laser radiolysis. For chemical restoration, aluminium and borohydride, tetraborate, hypophosphites, sodium citrate, formaldehyde, acetone, hydrogen peroxide and a lot of other organic and inorganic compounds are used. Methods of chemical condensation allow sufficient resistance against the aggregation of gold nanoparticles to be obtained.

The preparation of gold nanoparticles commonly involves the chemical reduction of gold salts in aqueous, organic, or mixed solvent systems. However, the gold surface is extremely reactive, and under these conditions, aggregation occurs. To circumvent this issue, gold nanoparticles are regularly reduced in the presence of a stabiliser, which binds to the surface and precludes aggregation via favourable cross-linking and charge properties.

Citrate, thiol-containing organic groups, encapsulation within microemulsions and polymeric coatings are used as stabilisers to passivate the gold nanoparticle surface. In particular, gold nanoparticles may be encrusted with biomolecules, with exciting prospects in biological sensing and imaging [20].

Several synthetic strategies exist, such as the two-phase liquid-liquid method initially described to create metal colloidal suspensions by Faraday in 1857 [21]. Faraday reduced an aqueous gold salt with phosphorous in carbon disulfide to obtain a ruby-coloured aqueous suspension of colloidal gold particles. According to the *Brust-Schiffrin method*[22] and its modifications [22,23], gold nanoparticles have been synthesised with numerous biomolecular coatings.

Using one of the above methods allows the appropriate type of gold nanoparticles with the set physical and chemical characteristics to be obtained. The most widely used in medicine known at the moment are spherical nanoparticles of gold in the dimensional range of *2 to 100 nm*; the most common method of achieving which is chemical recovery of water halide solution by the gold sodium citrate method proposed by Turkevich and Frens. Unique physical and chemical properties, such as inertia, stability, biocompatibility, the low level of cytotoxicity and others, cause significant medico-biological potential of this type of nanoparticles, especially at the level of cellular biochemical processes, and determine the prospects of their use as vectors for the targeted delivery of drugs.

We consider achieving the gold nanoparticles in our work to be described by the following reaction:

(1) $$\begin{array}{l} 2\mathrm{HAuC}l_{4}+5K_{2}CO_{3}\to 2\mathrm{KAu}O_{2}+5CO_{2}+8\mathrm{KCl}\\ \;+H_{2}O \end{array}$$

(2) $$\begin{array}{l} 2\mathrm{KAu}O_{2}+2СH_{3}\mathrm{СOС}H_{3}+K_{2}CO_{3}\to 2Au^{0}\\ \;+3СH_{3}\mathrm{СOOK}+\mathrm{KНC}O_{3}+{Н}_{2} \end{array}$$

The structure of gold nanoparticles synthesised by reaction (1) may be represented by the formula:

(3) $${\left\{m\left[Au^{0}\right]\mathrm{nAu}O_{2}{}^{-}:\left(n-x\right)K^{+}\right\}}^{-x}xK^{+},$$

where *m* is the number of molecules Au^0^; *n* is the number of excess ions AuO_2_^−^, firmly absorbed on the surface of the unit (usually *m* >*n*), which are potential forming; *x* is the number of ions within the diffusion layer; (*n*-*x*) is the number of counterions К^+^ absorbed at the layer. The number of potassium ions (*n*-*x*) less than the number of absorbed ions AuO_2_^−^ (*n*) results in the nanoparticle having a negative charge (*s*). The method used to obtain gold nanoparticles allows stable aqueous dispersions of nanoparticles of a certain size to be obtained.

**Figure 1:**
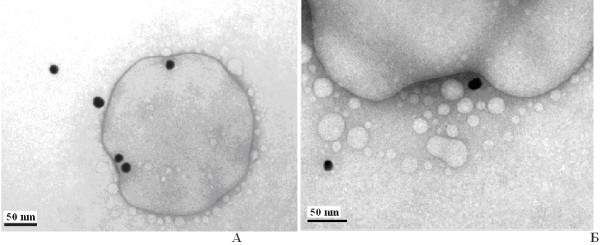
General view of AuNPs of discrete sizes: 10, 20, 30 and 45 nm.

A general view of synthesised AuNPs with discrete sizes 10, 20, 30 and 45 nm is presented in Figure 1. Gold nanoparticles are unique in terms of functionalisation (modification) of the surface, which is determined by their chemical properties: the ability to easily communicate with various ligand modifiers in soft conditions. The force of binding depends on the activity of donor-acceptor interactions in a ligand molecule, which can be carboxyl acids, amines, phosphines or thiols, and on the number of electron-donor sites in the ligand molecule that binds to the surface of the particle. Functionalisation of (option) the characteristics of nanoparticles, such as their size and surface, can significantly change the character of their influence on the biochemical processes of biological systems and the features of contact interaction with biological systems at the cellular level, microorganism levels and at the *in vivo* kinetics in general. This leads to the existence of considerable attention to the study on properties of compounds as a modifier and those modified (functionalised) with the use of gold nanoparticles: firstly on the toxic effect on cells and extracellular, membrane-related and intracellular biochemical processes, as well as studies on systemic impact on the organism as a whole (the response of the immune system and the state of biochemical indices of the blood and internal organs). Thus, Vallhov et al. point to the need for the absence of lipopolysaccharides on the surface of gold nanoparticles in the case of their application in medical practice [24]. The gold nanoparticles coated with alternately located subnanometre anion (sulfonate) and hydrophobic (bromide) in groups are able to successfully *pass through the plasma membrane* without destruction of the double layer, show high resistance to adsorption of the plasma protein and can be used to target the delivery of drugs into the cytoplasm, which opens up new prospects in approaches to the modification (functionalisation) of nanoparticle surface [25].

Thus, the physicochemical properties of gold nanoparticles, the key of which are the size, form, charge and structural peculiarities of molecules on the modifier surface, significantly influence the efficiency and biochemical mechanisms of the interaction of gold nanoparticles with biological systems at different levels of the organisation - from prokaryotic cells to the microorganism, especially at the level of cellular biochemical processes.

#### Peculiarities of the interaction of gold nanoparticles with biological systems and their influence on molecular and biochemical processes

The behaviour of nanoparticles in contact with biological systems at different levels of the organisation, their stability and internal and extracellular distribution are not similar and essentially depend on the size, composition and morphology of nanoparticles. At the level of the macrobiosystem, there is a dependence of the interaction of nanoparticles with biosystems on the methods of their introduction into the body (intravenously, subcutaneously and intramuscularly). In addition, the arrival of gold nanoparticles at the target destination in contact with biological systems is connected to crossing the organism's number of protective barriers, mainly the cell membrane and reticuloendothelial system.

Different routes of administration can result in various effects on the biodistribution of drug carriers. Injected intravenous gold nanoparticles get into the vascular system and are *distributed to organs and peripheral tissues of the body*. In the bloodstream, they come in contact with blood cells, platelets and coagulation factors and plasma proteins. Serum proteins can absorb or opsonise nanoparticles. It should be noted that under the conditions of intravenous injection, nanoparticles are fairly quickly eliminated from the blood circulation, to a large extent by macrophages of the liver (Kupffer cells) and spleen (border zone and the red pulp). Data show that the hydrodynamic diameter of the particles and their physicochemical characteristics affect their clearance from the blood and, consequently, the elimination half-life of nanoparticles in the blood, and with the whole body, the diameter of the particles is inversely proportional to the speed of glomerular filtration and directly connected with their half-life in the flesh and blood.

The role of endothelial cell monolayer vessels, which act as a dynamic, semipermeable barrier that regulates the transport of liquids, molecules and particles between intravascular and extravascular space, should be noted. In the case of normal, intact endothelium, nanoparticles with size less than 5 nm can quickly get to the extravascular space [26]. In the opinion of the Moghimi et al., very small nanoparticles (1 to 20 nm), especially in the conditions of long circulation, can slowly penetrate from vessels in the fabric of space, from where they are transported through the lymph vessels to the lymph nodes [27].

**Figure 2:**
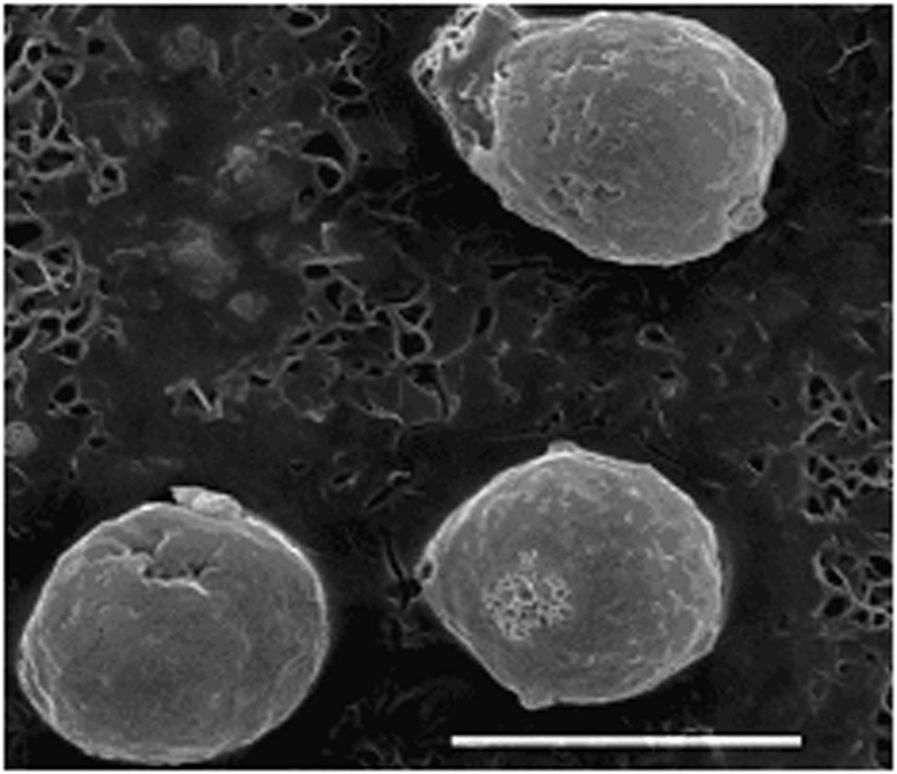
Electron microscopic images of cell line U937 (×3,600).

**Figure 3:**
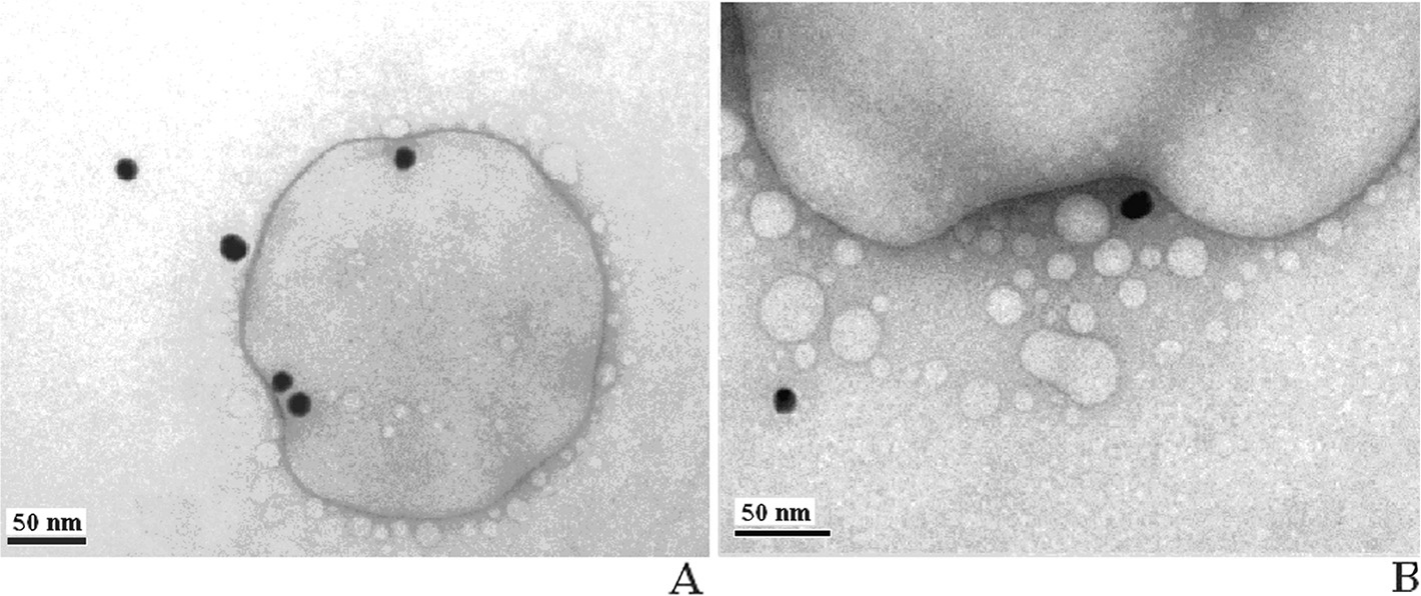
**Electron microscopic image of intracellular localisation of gold nanoparticles in the vacuole cell line U937.** Nanoparticle size: 20 (**A**) and 10 nm (**B**). The cell concentration is 106 cells/ml of aquatic macrophytes in frozen storage buffer (FSB) within 3 to 5 min with gold nanoparticles (final concentration is 12.7 g/ml for metal).

**Figure 4:**
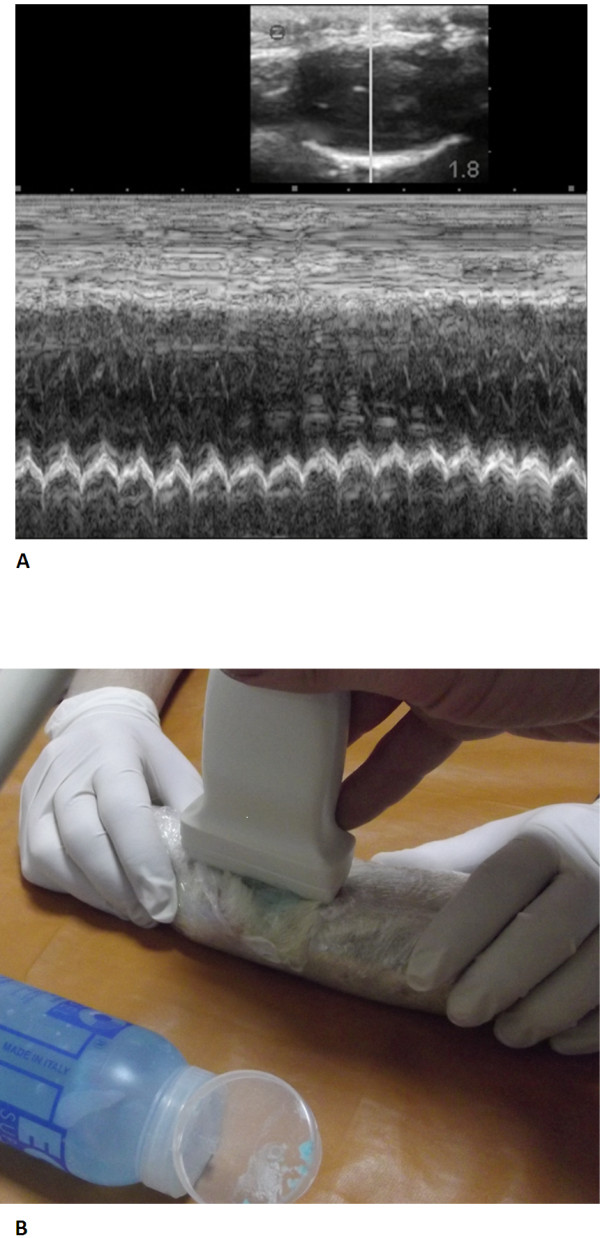
**Electron microscopic image of intracellular localisation of 30-nm AuNPs in cell line U937 lysosomes.** The cells in the final concentration of 106 cells/ml of aquatic macrophytes in the FSB buffer within 3 to 5 min with gold nanoparticles (final concentration is 12.7 g/ml for metal).

**Figure 5:**
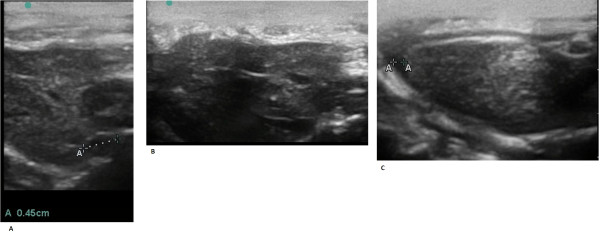
**Confocal microscopy images.** Scanning on the *z*-axis at intervals of 1 mm. Incubation of cells for 3 min with the drug nanoparticles. Accumulation of 30-nm gold nanoparticles in the culture of eukaryotic cells.

The influence of nanoparticle size on the nature of their interaction with biological systems is illustrated by the work of Praetorius and Mandal, who noted that nanoparticles *less than 20 nm can freely penetrate the wall of the blood* vessels, and the small size allows these nanoparticles to be delivered not only intravenously but also locally intramuscularly or subcutaneously [28] under imaging guidance, which inspires new directions for imaging-guided drug delivery systems with application of sonoporation. The electron microscopic images of AuNPs of different sizes interacting with cell line U937 are presented in Figures 2, 3, 4; accumulation of 30-nm gold nanoparticles in the culture of eukaryotic cells is demonstrated in Figure 5.

Data on the molecular-biochemical effects of gold nanoparticles of different sizes are quite limited and fragmented. The size of the nanoparticles can be compared with the size of biomolecules - components of eukaryotic and prokaryotic cells. The dimensional range of gold nanoparticles (1 to 100 nm) stipulates a number with special chemical properties and unique character of influence at the molecular-biochemical level. Thus, the gold nanoparticle average size of 9 nm inhibits the activity of cytochrome P450 isoenzymes (SRM 450 1A2, SUR 450 2C9, SUR 450 2C19 and SUR 450 3A4). The mechanism of inhibiting the impact of nanoparticles on the activity of the hemeprotein should be clarified; however, it is connected with the physicochemical characteristics of these nanoparticles and the hydrophobic environment heme molecule P450 that provides the possibility for gold nanoparticle to compete with the enzyme substrate.

Gold nanoparticles of 2-nm size, functionalised by 2-malonic acid (10-mercapto decanol), have chaperone-like properties and are able to influence the folding of proteins and fіbrіlogenesis. The chaperone-like properties of gold nanoparticles open up new prospects in the creation of drugs, including those for the treatment of brain diseases.

The conjugation of gold nanoparticles with plasmid and DNA nucleotide sequences increases the level of their penetration in eukaryotic cells. Conjugates, obtained in physiologically acceptable conditions, are able to pass through the membrane without damaging and destructing it. This opens new perspectives both for fundamental research in the field of molecular biology and genetics as well as for application to problems in the development of diagnostic tools. The resulting gold nanoparticles have biological applications, for instance, in the detection of polynucleotides via hybridisation to oligonucleotides appended on the nanoparticle surface [29].

The research on pharmacological, biochemical, physicochemical and colloidal-chemical mechanisms of interaction of nanoparticles with biological objects (cells of the macro- and microorganism) will allow not only the establishment of their positive or negative effect on biostructure and the surrounding world but also the possibility of wide application in medicine as effective drugs, as the media for targeted drug delivery and physiologically active substances up to the pathological process.

*Oxidative stress* is one of the main factors in cellular ageing and other cellular disorders [30]. While therapeutic treatments cannot be based exclusively on the abatement of oxidative stress, neutralising this cellular disorder could minimise collateral damages associated with the transformation of biomolecules in the cytosol. Traditionally, *reactive oxygen intermediates* were considered to be toxic by-products of aerobic metabolism, which were disposed of using antioxidants.

Superoxide radicals and hydrogen peroxide [31] balance, together with sequestering of metal ions, is thought to be important to prevent the formation of the highly toxic hydroxyl radical via the metal-dependent *Haber-Weiss* or the *Fenton* reactions. Also, gold nanoparticles have been used in a model of diabetes showing an antioxidant effect [32]. Considering the ability of gold to trap carbon-centred radicals as well as to decompose hydroperoxides [33,34], Au/CeO_2_ has some antioxidant activity against cellular oxidative stress.

#### Coactivity with cerium dioxide nanoparticles

A significant number of reports describe that *cerium oxide nanoparticles* (CNPs), either pristine or surface-modified with PEG or biopolymers, are biocompatible and that they exhibit properties as superoxide dismutase to decompose reactive oxygen species (ROS) generated under oxidative stress in cells [35-38]. Alili et al. reported that nanoceria downregulates both the expression of alpha-smooth muscle actin-positive myofibroblastic cells and the invasion of tumour cells. Furthermore, concentrations of nanoceria being nontoxic for normal (stromal) cells show a cytotoxic effect on squamous tumour cells. Treatment with redox-active CNP may form the basis of stromal cell protection from the dominating influence of tumour cells in tumour-stroma interaction, thus being a promising strategy for chemoprevention of tumour invasion [39]. The application of redox-active CNP may form the basis of new paradigms in the treatment and prevention of cancers [40].

We found [41] that synthesised nanocrystalline cerium has antibacterial activity *in vitro* against different groups of opportunistic microorganisms: clinical strains of *Escherichia coli*, *Staphylococcus aureus* and *Candida albicans*[41]. Antiviral activity of nanosize cerium dioxide sols in animal cell culture has been studied [42]. The inhibiting effect of the mentioned sols against the reproduction of vesicular stomatitis test virus was demonstrated for the first time in the case of a preliminary 24-h contact with cells lines L929 and EPT. The effectiveness of protective action depends on the initial precursor and the way of obtaining the water nanosize nanosols. Ceria-supported gold nanoparticles exhibit peroxidase activity and act as radical traps. The antioxidant activity of Au/CeO_2_ against ROS is demonstrated by studying the cellular behaviour of Hep3B and HeLa in a model of cellular oxidative stress. It is determined that Au/CeO_2_ exhibits higher antioxidant activity than glutathione, the main cytosolic antioxidant compound, and its CeO_2_ carrier.

Au/CeO_2_ is a highly active heterogeneous catalyst for many oxidation and reduction reactions, and there are abundant literature data describing its preparation and characterisation [43]. Thus, using well-established nanoparticulated gold catalysts has a large potential with remarkable biocompatibility in cellular biology.

Menchón et al. [43] described the antioxidant activity of Au/CeO_2_ that is about 20% higher than the conventional glutathione antioxidant and is notably efficient for high ROS concentration. The authors suggest a way to use ceria nanoparticles as biocompatible carrier and Au/CeO_2_ as biocatalyst in other processes. Further studies are necessary to assess whether the decrease in ROS concentration caused by Au/CeO_2_ does not produce transformation of biomolecules in the cell [43].

#### Antiangiogenesis

However, the results of Tsai et al. suggest that nanogold has therapeutic potential in ameliorating *rheumatoid arthritis* and may be applicable to the modulation and inhibition of various vascular endothelial growth factor-dependent chronic inflammatory diseases [44]. Authors have shown that intraarticular delivery of nanogold is an effective treatment strategy for *collagen-induced arthritis* since nanogold binds strongly to thiols and amines [17,45], inhibits vascular endothelial growth factor (VEGF)165-induced endothelial cell proliferation by interacting with sulphur/amines present in its heparin-binding domain and thereby inhibits VEGF165-induced signalling [46,47].

Thus, nanogold has antiangiogenic effects; it may be beneficial for the treatment of arthritis. Moreover, nanogold inhibits VEGF-induced permeability in models of ear tumour and ovarian tumour in mice [46].

### The clinical applications of gold nanoparticles

#### Diagnostics

Gold nanoparticles are used to detect biomarkers in the diagnosis of heart diseases, cancers and infectious agents (e.g., home pregnancy test) [47]. A pioneering work toward an assay of *Alzheimer diseases* using AuNPs has been firstly reported by Van Duyne's group [48,49]. Multivalent AuNPs were found to inhibit *HIV fusion*[50]. Successfully prepared AuNP probe for *hepatitis B* virus DNA could be potentially applied to multi-gene detection chips [51]. A successful application of the AuNP nanoprobe by Baptista et al. was the sensitive detection in clinical samples of *Mycobacterium tuberculosis*[52]. Diabetes was characterised as a multifactorial disease using the AuNP nanoprobe method mentioned above which involved capturing the analyte with a magnetic particle featuring recognition elements followed by binding of a AuNP with a second recognition agent and marker DNA strands for *cancer detection*[53].

#### Therapeutic agent delivery

Nanotechnology will assume an essential place in drug delivery and human therapeutics. A wide variety of nanoparticles exist already, and diverse methods of synthesis have been developed [20] and are applicable for personalised medicine in the future [10]. Over the past decade, several delivery vehicles have been designed based on different nanomaterials, such as polymers [54], liposomes [55], nanotubes [56] and nanorods [57].

Therapeutic agents can also be coated onto the surface of gold nanoparticles. The large surface area/volume ratio of gold nanoparticles enables their surface to be coated with hundreds of molecules (including therapeutics, targeting agents and anti-fouling polymers) [58].

Therapeutic vectors carry drugs, genes and imaging agents into living cells and tissues [59]. The drug vectors should also be stable in the circulatory system yet become labile under appropriate conditions when the targeted organ is reached. The drug vectors carry the drug by encapsulation or, more or less, strong binding (covalent, coordination or supramolecular bond) [11].

The pharmacokinetic parameters of these nanoparticles may be altered according to size, shape and surface functionalisation [11,20,47]. Careful design of nanoparticle delivery agents will result in successful localisation and drug delivery to specific biological targets coupled with the efficient evasion of the reticuloendothelial system. Different routes of administration can result in various effects on the biodistribution of drug carriers.

Moreover, nanoparticles can be used to alter the kinetic profiles of drug release, leading to a more sustained release of drugs with a reduced requirement for frequent dosing. Particularly interesting applications of nanoparticles in drug delivery relate to the *central nervous system* and the *cardiovascular system*.

##### The basic requirements for medicines for delivery systems

New methods of drug and medicine delivery create a new niche in the pharmaceutical market. If in 2006, more than 30 companies engaged in the development and production of nanoparticles for delivery of medicines, in 2011 this amount had risen to more than 70 [60,61]. Despite a number of advantages, in the system of medicine delivery, there are certain deficiencies related to the difficulty in controlling the synthesis of the media from batch to batch. Earlier, the problem on the stability of the nanomaterials was also mentioned. Special attention should also be given to the toxicological aspects of using nanoparticles, which is still insufficiently studied. Note that nanomedicines should not be *immunotoxic*. It is known that the reaction of the immune system to nanoparticles depends on their *size*[11,47]. In particular, nanoparticles with a diameter of 200 nm more strongly activate the complement system and are quickly eliminated from the circulatory system.

It is also believed that decreasing the diameter of nanoparticles increases its toxicity by increasing the specific surface. This, in turn, leads to the activation of oxide recovery processes, in which there is participation of the atoms of nanoparticles and the formation of free radicals. There is also the problem of uncontrolled self-assembly and the need for control over the functions of nanoparticles, which are used for drug delivery [60,62].

The following are the requirements for the nanomaterials [63]:

•The therapeutic effect being compared to that of a similar product which is used in medical practice must be pronounced.

•The product should cause fewer side effects than a similar drug.

•It must be stable and maintain the chemical structure in the course a certain time.

•It must not adversely affect the clinical pharmacological properties of the preparations, which are used in medical practice.

•The pharmacoeconomic indicators of nanomaterials should be positive.

•The dosage form of nanomaterials should be convenient for use.

•It is also preferable that the technology of nanomaterial production be available and environmentally clean.

##### Size-related tissue permeation of gold nanoparticles

The size of the AuNPs, which are currently being studied and applied in biology and medicine, varies from 1.0 to 2.0 nm (nanoparticles used as X-ray contrast agents) to shell structures with the size of 50 to 500 nm [11]. In practice, the useful size of nanomedicines more normally falls within the range of 5 to 250 nm as this tend to have a similar range of properties based on physiological and anatomical consequences [64].

Different routes of administration can result in various effects. Permeation of gold nanoparticles through the skin and intestine was found to be size dependent [65]. *In vivo* distribution largely depends on the particle size and surface properties such as surface charge and surface hydrophobicity [66,67]. Sonavane et al. [65] established organ-specific delivery relating to NP sizes. According to their data, gold NPs of 15-nm size are not cumulated in the pancreas; 50-nm-size gold NP cumulates in the liver, lung and spleen tissues; 100-nm-size gold NP also showed higher accumulation in the liver, lung and spleen; and 200-nm-size gold NPs accumulate in the liver followed by the spleen, lung and kidney. Two hundred-nanometre gold NPs show a very short presence in organs including the blood, brain, stomach and pancreas and are more quickly eliminated from the circulatory system [11,47].

The *blood–brain barrier* (BBB) is a formidable challenge for many therapeutic agents [65]; nanotechnology may breach this barrier and establish a new frontier for neuropharmacologic agents. Several hypotheses exist for gold NP permeation through the BBB. Nearly 100% of the surface area of the capillary basement membrane is covered by the end-feet of processes originating from brain astrocytes, and these astrocytic end-feet are separated from the capillary endothelium by a distance of only 20 nm. Gold NPs with sizes of 15 and 50 nm were able to pass the blood–brain barrier, as evident from gold concentration in the brain [68]. Hence, smaller-size gold NP may transfer through these gaps, making possible the delivery of entrapped activities into the brain parenchyma without inducing BBB permeability alteration [69].

It has been shown qualitatively (by light microscopy) and quantitatively in a series of works that persorption of metallic iron particles with diameters ranging from 5 to 110 mm can enter the body by means of paracellular uptake by the lymph, blood and other bodily fluids [70-73]. Nanoparticles smaller than 20 nm can freely penetrate the wall of blood vessels [28].

##### Sonoporation

Vibration caused by ultrasonic waves can change the structure of the cell membrane and enhance its permeation. In the last decade, a new ultrasound-aided method, sonoporation, has been proposed and utilised to transmit target molecules (such as drugs and DNA) into cells for therapy [11]. However, since nanoparticles less than 20 nm can freely penetrate the wall of blood vessels [29], sonocavitation has to extend the size of the delivered particles and increase the amount of transported NPs to the cells and mitochondria by creating pores in cellular and subcellular membranes, providing simultaneous imaging. Yu-Hsin et al. [63] have shown an approximately 60% improvement in terms of fluorescence signals from the cellular uptake of gold nanoparticles after sonoporation treatment. Our recent results demonstrated that polyplex gene transfer by ultrasound (US) exposure is effective and illustrated the potential of ultrasound-triggered gene delivery technology for gene therapy [74]. Therefore, we conclude that controlled release is feasible and can further improve the therapeutic effects of nanoparticles.

##### Optoacoustic imaging

In the application of AuNPs in diagnosis, optical signals are important not only. An interesting study was conducted by Eghtedari et al. (2007) [75]. The authors suggest optoacoustic method in the diagnosis of cancer. The method is based on the ability of AuNPs under the influence of near-infrared light to allocate heat. It in turn can be passed around tissues and turned into sound waves that may be registered by an ultrasound receiver. Thus, researchers combine the high selectivity of gold nanorods and antibody bioconjugates and high-resolution ultrasound examination. An optimal configuration for gold nanorod (GNR)-enhanced optoacoustic imaging was experimentally determined, demonstrating in particular its feasibility with a conventional echographic device. The proposed approach can be easily extended to the quantitative performance evaluation of different contrast agents for optoacoustic imaging [76].

##### Contrast media

Numerous plasmonic NPs have been recently developed and tested as potential contrast agents (CAs) for optoacoustic imaging [77]. In most cases, gold is the metal of choice due to its high stability, facile chemistry and easy bioconjugation [78,79] as well as its generally benign toxicity profile [80]. Various types of gold NPs have been experimentally tested as optoacoustic CAs, such as nanospheres [81-83], nanoshells [83] and nanocages [84], but the class of NPs most significantly explored for optoacoustic imaging applications is represented by GNRs [85,84].

#### Treatment

Gold nanoparticles are being investigated as carriers for drugs [11,54-59]. Kogan et al. utilised AuNPs in weak microwave fields in order to dissolve amyloid aggregates [85]. The utility of gold nanoparticles for diagnostics [11] and cancer treatment [86] were reported. The methodology of treatment in the application of AuNPs is the phenomenon of surface plasmon resonance. As noted above, nanoparticles can absorb light of certain wavelengths and convert its energy into localised heat.

High catalytic activity is another gold property, which appears at the nanoscale. It is associated with a large number of gold surface atoms, which interact with the substrate. Certain techniques use the catalytic activity of AuNPs. Gold combined with cerium oxide catalyses the oxidation of carbon monoxide to carbon [87].

##### Theranostics

The application of nanoparticles allowing the combination of therapy and diagnosis, known as *theranostic*, has received increasing attention in biomedicine [88].

##### Photodynamic therapy

Near-IR absorbing gold nanoparticles (including gold nanoshells and nanorods) produce heat when excited by light at wavelengths from 700 to 800 nm. This enables these nanoparticles to eradicate targeted tumours [89,90]. When light is applied to a tumour containing gold nanoparticles, the particles rapidly heat up, killing tumour cells in a treatment also known as hyperthermia therapy.

Gold nanoparticles are used in a variety of sensors [91] and probes. Gold nanoparticles also scatter light and can produce an array of interesting colours under dark-field microscopy. The scattered colours of gold nanoparticles are currently used for biological imaging applications [92] in transmission electron microscopy. For instance, antibody-modified AuNPs displayed a million-fold higher sensitivity to the detection of prostate-specific antigen [93].

### Perspectives for predictive, preventive and personalised medicine

Thus, the applications of personalised nanomedicine in novel disciplines that concern cancer (nano-oncology) [87], neurological disorders (nanoneurology), cardiovascular disorders (nanocardiology) [94,95], diseases of the bones and joints (nano-orthopaedics), diseases of the eye (nano-ophthalmology), nanoendocrinology [32], nanoimmunology and infectious diseases [7] can be distinguished.

#### Nanocardiology

The rapid development of nanomedicine has not bypassed cardiovascular diseases. Although the publications in the sphere of nanomaterial use are still quite few, there are already attempts to use nanoparticles as vectors in targeted delivery of cardioprotective drugs. Gold nanoparticles are known to activate metabolic processes, reduce blood pressure, improve blood circulation and have expressed bactericidal effect. Nanobiotechnology approach has the potential to improve the results of cell therapy for myocardial infarction, which is on clinical trials currently [5]. It was reported that targeted imaging and therapy applications with perfluorocarbon nanoparticles are relevant to a broad spectrum of cardiovascular diseases, ranging from asymptomatic atherosclerotic disease to acute myocardial infarction or stroke [5]. Perfluorocarbon nanoparticles provide an opportunity for combining molecular imaging and local drug delivery in cardiovascular disorders. Utilising targeted perfluorocarbon nanoparticles has been demonstrated for a variety of applications in animal models including the diagnosis of ruptured plaque, the quantification and antiangiogenic treatment of atherosclerotic plaque and the localisation and delivery of antirestenotic therapy following angioplasty.

Nanoscale particles can be synthetically designed to potentially intervene in lipoprotein matrix retention and lipoprotein uptake in cells - processes central to atherosclerosis. Nanoengineered molecules called *nanolipoblockers* can be used to attack atherosclerotic plaques due to raised levels of low-density lipoproteins [96]. An experimental study in rats using injectable self-assembling peptide nanofibre bound to platelet-derived growth factor demonstrated sustained delivery to the myocardium, resulting in decreased cardiomyocyte death and preserved systolic function after myocardial infarction [97]. In studies on rats, cell therapy with insulin-like growth factor 1 delivery by biotinylated nanofibres improved systolic function after experimental myocardial infarction [98]. As various mechanisms enabling cardiac regeneration are becoming elucidated, novel technologies using degradable microspheres for controlled release systems and self-assembling peptide nanofibres for cell and factor delivery were reported [99].

##### Nanoimmunology

Cardiovascular diseases are strongly connected to immune response. Pathogenesis of cardiovascular diseases is associated with the dysfunction of cytokine production. In most autoimmune diseases, a stereotyped response is observed in the form of a large subpopulation of activated Th1 lymphocytes [100] not rarely observed, decrease in the number of T lymphocytes, impaired T helper/suppressor ratio downward suppressor activity and weakening response to mitogens. In patients with autoimmune disease, often increased levels of proinflammatory cytokines (TNF-α, IL-1, IFN-γ) may result in the aberrant activation of the innate immune response [101]. During a persistent heart muscle damage, exposure of the intracellular content to dead cells activates the innate immune response, such as the activation of Toll-like receptors (TLR). In the heart, TLR2 and TLR4 are perhaps involved in the host response to myocardial infarction [102]. The activation of TLR initiates the imbalance of TLR-induced cytokines. We hypothesise that AuNPs may affect the calcium channels and impact the imbalance of cytokines.

##### Nanoneurology

Kogan reported the use of local heat delivered by metallic nanoparticles selectively attached to their target as a molecular surgery to safely remove toxic and clogging aggregates, particularly the amyloid beta protein involved in Alzheimer's disease, a neurodegenerative disease [86]. We hypothesise that due to NP bioeffects against cellular oxidative stress, targeted theranostic treatment for neuromuscular diseases (myopathy, neuropathy, latent trigger points) may be applied in the near future after approval by evidence-based studies [103]. Combination with targeted biological therapies such as the growth factor of platelet-rich plasma [104] gives new opportunities for neuromuscular disease management.

Nanoneurosurgery is a conceptual leap necessary for neuroscientists as well as neurosurgeons in developing and applying nanotechniques to neurosurgery at the nano level. According to Andrews et al. [105], nanoscaffolds offer mechanical enhancement of neurorepair; carbon nanotube electrode arrays can provide nanolevel electrical and chemical enhancement. Even the traditional ‘cut-and-sew’ surgery is being taken down to the micron, if not nano, level for single axon repair, and the technology can use capillaries to deliver therapeutics to virtually any portion of the nervous system with greater-than-pinpoint accuracy.

Future calls for upcoming PPPM-related studies with particular applications of AuNPs for therapeutic drug delivery properties for multifunctional nanomedical solutions related with genetics and cell biology are required in the following fields:

•Nanohepatology

•Nanonephrology

•Nanoallergology

•Nanogastroenterology

### Reliable cardiovascular animal models

As the cardioprotective properties of gold nanoparticles are still not conclusively confirmed, particularly for heart failure as well its role in drug delivery, that calls for study in a reliable model. In cardiovascular research, animal models have allowed the study of cardiovascular disease in the early stages, as well as the investigation on the mechanisms of the pathogenesis of cardiovascular disease and the effects of drug intervention. Doggrell et al. [106] suggest that an ideal animal model for any cardiovascular disease in humans should follow five characteristics: (1) mimic the human disease; (2) allow studies in chronic, stable disease; (3) produce symptoms which are predictable and controllable; (4) satisfy economical, technical and animal welfare considerations; and (5) allow measurement of relevant cardiac, biochemical and haemodynamic parameters. As cardiovascular disease is uncommon in young humans but markedly increases with age [107], age-related changes of an animal should be considered.

However, existing models focus mostly on local heart function assessed by echocardiography, and the dynamic *in vivo* examination of systemic circulation of the animals was not sufficiently evaluated. Without the use of visual navigation methods, injection methods are still limited by introduction of agents orally, into the tail vein, intraperitoneally and subtentorially. We did not find any data regarding the use of precision injection under US guidance for rat model of heart failure.

**Figure 6:**
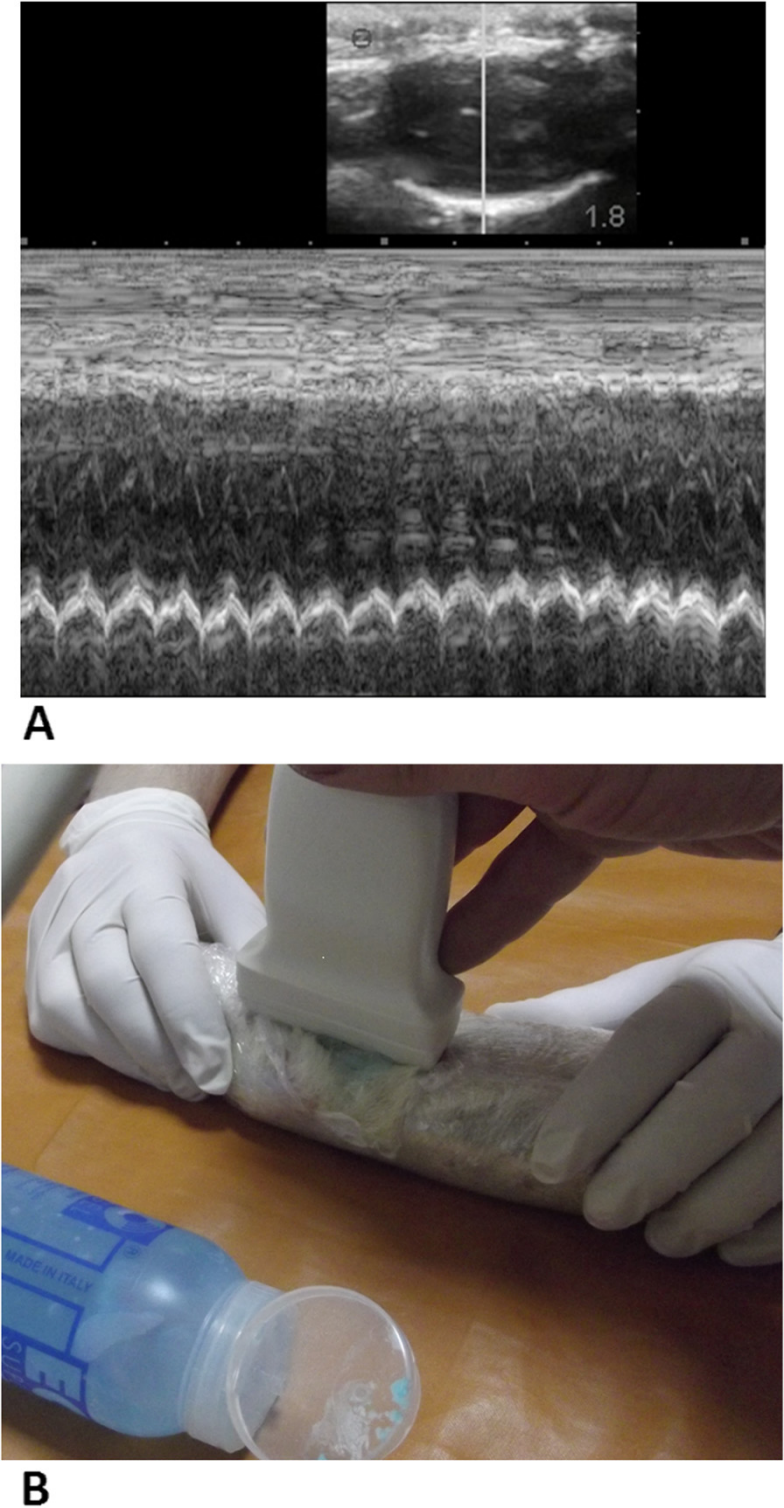
**Ultrasound survey of an immobilised rat (A) and echocardiography (B).** After [109].

**Figure 7:**
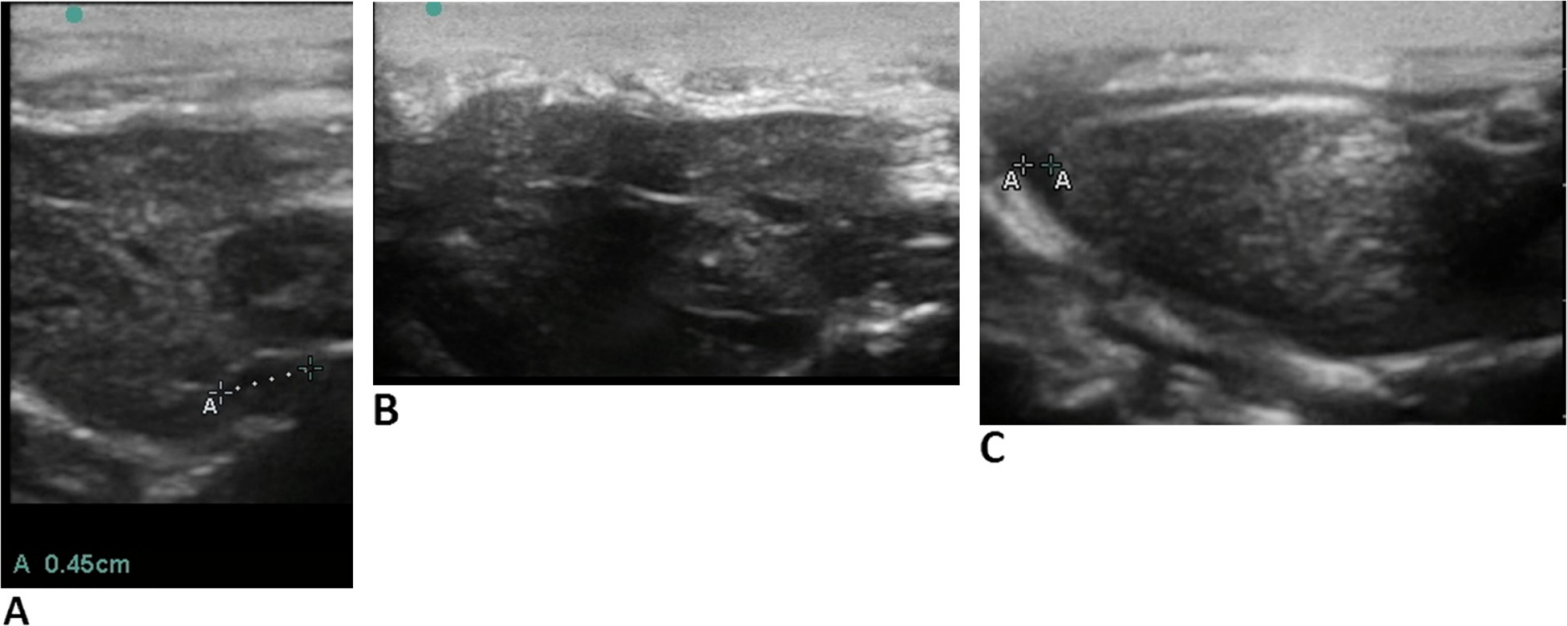
**Ultrasonogram of a rat with congestive heart failure.** (**A**) The inferior vena cava expanded to 4.5 mm. (**B**) Dilated hepatic veins - indirect signs of venous congestion in a large circulation. (**C**) Mild ascites in a rat. A strip of liquid is revealed near the liver - an indirect sign of venous congestion in a large circulation [109].

**Figure 8:**
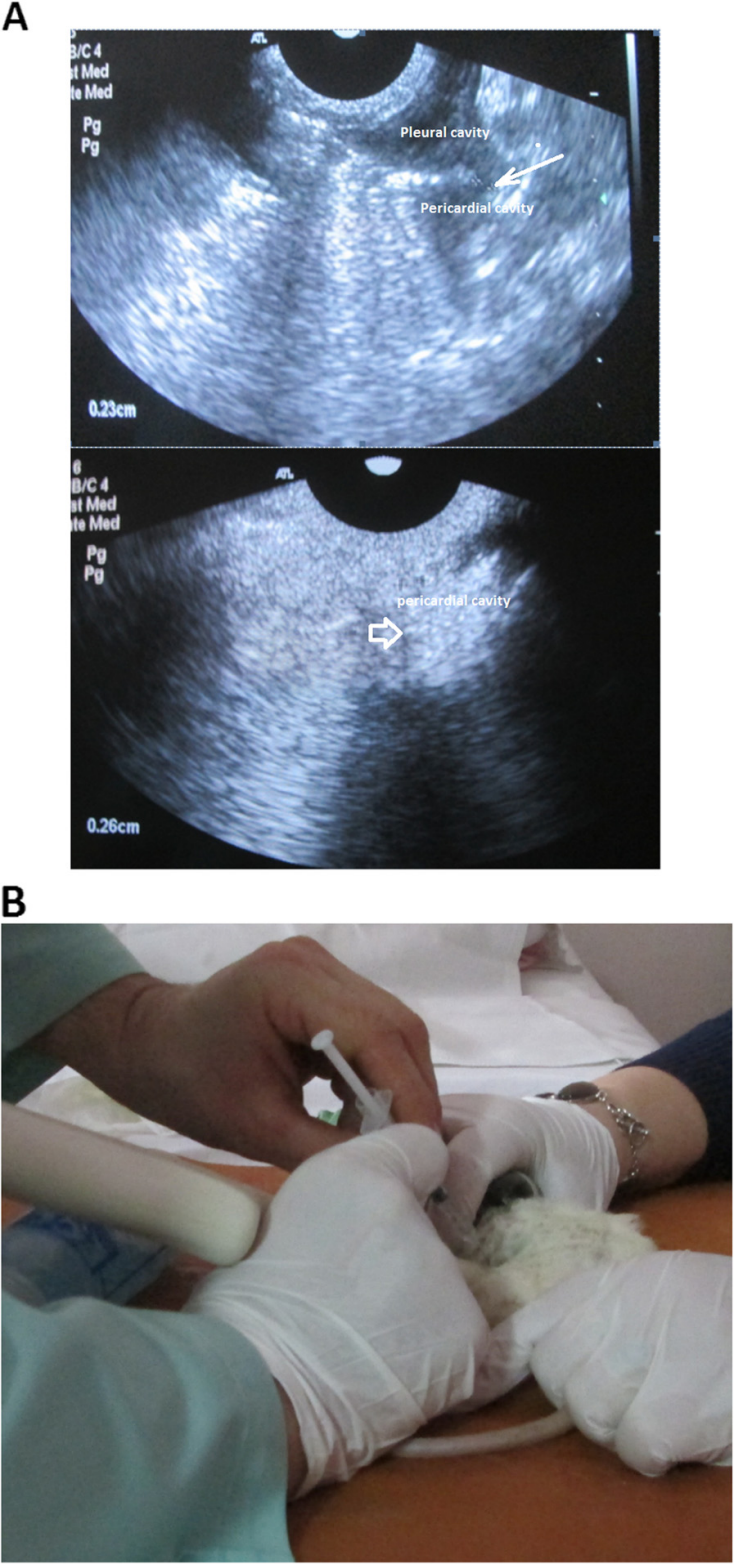
**Ultrasound guided injection in a rat.** Injecting into the pleural cavity (**A**) and spreading to the pericardial cavity (**B**) through the pore (thin arrow); the big arrow indicates fluid in the pericardial cavity [109].

Recently, we performed a study describing the use of general-use US equipment to study *in vivo* a novel medicine testing in mice [108] and described and patented the method of doxorubicin heart failure rat model using general-use US equipment focusing on peripheral circulation assessment (Figures 6, 7, 8). The suggested optimal cardiotoxic dose of doxorubicin for extended and longitudinal observation of rats has not been determined [109].

### Assessment of nanomaterial risks

Particular attention in the context of medical use should be given to the toxicity of nanogold. Nanotubes, because of their surface properties and very small size, may bind and transport toxic chemical compounds and be toxic themselves by generating free radicals [102], inducing oxidative stress, and this is a disadvantage for their application in medicine [110]. Seaton et al. established potential factors of nanoparticle toxicity [111], which include length (greater than 15 μm - below it, the fibre can be removed by pulmonary macrophages), diameter (less than 3 μm - allows fibres to be inhaled into the gas-exchanging part of the lung), insolubility, resistance to dissolution in the lung environment and sufficient dose of delivery to the target organ. A number of studies indicate that AuNPs have low cytotoxicity [15] and high biocompatibility. Despite this, there is insufficient research on the toxicity of nanogold *in vivo*, which is a necessary step before clinical re-housing of drugs in AuNPs [7,112,113]. The Au/CeO_2_ complex showed remarkable biocompatibility as demonstrated by measuring cellular viability, proliferation and lack of apoptosis for two human cell lines (Hep3B and HeLa) [43].

#### Cytotoxicity

The long history of (almost legendary) gold colloid use for therapeutic purposes suggests that AuNPs should be biocompatible. The potential of AuNPs in nanomedicine, especially for imaging, diagnostic and therapy, however, requires their toxicity to be thoroughly examined with maximum care and accuracy. The cytotoxicity of AuNPs has been examined and reviewed by several research groups [114]. Since everything is toxic at a high dose, the important question is whether AuNPs are toxic at the concentration at which they will be used, believed to be in the range of 1 to 100 AuNPs per cell. Also, *in vivo* conditions are different from *in vitro* results, and in particular, more *in vivo* studies are called for. Thus, no general conclusion can be drawn at present. It has been suggested, however, that it could be applicable to use AuNPs as reference nanoparticles for low cytotoxicity in the setup of a nanoparticle toxicity scale, given the higher toxicity of carbon nanotubes and quantum dots compared to non-cationic AuNPs. Finally, AuNPs are active redox and therefore reduce the production of reactive oxygen and nitrite species [114].

For given physicochemical and biochemical parameters, the most pronounced toxic effects on biological systems are characterised by different amounts of 10-nm gold nanoparticles. Gold nanoparticles with sizes of 30 and 45 nm have the most biocompatible biological nature, indicating the prospects of their use as vectors in targeted cardiotherapy.

Duffin et al. showed that the surface area metric drives the overload response. The extent of inflammation was demonstrated as being a function not of the mass dose instilled but interestingly of the surface area dose instilled. Since low-toxicity nanoparticles present a ‘special’ case of high surface area, they are relatively inflammogenic [115]. Thus, to the toxicity survey, it appears that AuNPs usually show rather little toxicity, if any, because many cytotoxicity studies report negative cytotoxicity finding results.

## Endnotes

Considering the data reviewed above, pharmacological, pharmaceutical and toxicological aspects of the application of gold nanoparticles in biomedical purposes still remain poorly understood. Thus today, there is an actual in-depth study on the mechanisms of action of new drugs on the basis of nanoparticles and their side effects and the development of pharmaceutical technologies, obtaining adequate dosage forms to be successfully used in medical practice. We consider the aim of upcoming work (project) to develop fundamentally new science-based approaches to design biosafe and biocompatible nanoconstructions based on gold nanoparticles for targeted delivery of cardiotropic agents.

### The goals for the next study

The following are goals for the next study:

1. To develop new evidence-based approaches for the synthesis of biologically safe and biocompatible gold nanoparticles and the creation of nanoconstructions based on gold nanoparticles and cardiotropic drugs to improve their delivery for cardiovascular pathologies

2. Identify biosafety, biocompatibility and biological effectiveness of nanoconstructions created from gold nanoparticles and the cardiotropic drug Simdax (‘AuNPs-Simdax’ conjugate)

3. Conduct preclinical trials of the developed AuNPs-Simdax conjugate in experimental heart failure in animals, assessing the suggested rat model for application effects and comparing with Simdax - a proven medication for congestive pleural effusion- using general US equipment

4. To test sonoporation effect to increase nanoparticle delivery into myocardial cell in rats

The stages for the implementation of the project are as follows:

•Development of original protocols for colloid-chemical synthesis of gold nanoparticles of different sizes

•Determination of physicochemical and biological characteristics of the synthesised gold nanoparticles in their interaction with biological systems at different levels of the organisation

•Evaluation of the distribution of gold nanoparticles in the bodies of the laboratory animals in case of intravenous and imaging-guided administration

•Development of scientifically based approaches to design conjugate based on biosafe gold nanoparticles

•Definition of features of gold nanoparticle and model protein conjugates and study the aggregative stability of gold nanoparticles in model systems of blood

•Establishment of immunogenic properties of AuNPs and AuNPs-Simdax conjugate in the experimental model

•Determination of the targeted delivery of AuNPs and AuNPs-Simdax conjugate to myofibrils of cardiac muscle in experimental models

•Assessment of the impact of AuNPs and AuNPs-Simdax on the myofibrils of the heart muscle

•Definition of hypoxic, infarction changes at the morphological and ultrastructural level

•Definition of the biosafety of AuNPs and AuNPs-Simdax conjugate

•Determination of the toxicity and effectiveness of AuNPs and AuNPs-Simdax

•Determination of the impact of AuNPs and AuNPs-Simdax on the immune resistance of organism

•Optimising the design of AuNPs and AuNPs-Simdax in the context of clinical trials

•Conduction of preclinical trials of AuNPs and AuNPs-Simdax

•Testing sonoporation effect to increase nanoparticle delivery into myocardial cell in an animal model

### Consolidation of the PPPM concept

#### Personalised medical approach

Imaging/sonoporation combined with direct visualisation of target tissues and optoacoustic phenomena to detect nanoparticles *in vivo* and its potential to be a contrast agent for US/MRI imaging is a significant opportunity for personalised theranostics.

#### Predictive medical approach

Optoacoustic phenomena are a relevant basis for contrast imaging with biomarker registration with high predictive value potential. Extensive application of sensor based on AuNPs allows us to think about developing novel technologies for minimally invasive diagnostic/treatment procedures.

#### Preventive medical approach

Strong antioxidative effects combined with high biosafety are a crucial challenge in anti-ageing strategy. Further studies are necessary to clarify the molecular mechanisms of AuNP effects and its relevant dosage.

### Future outlooks and recommendations

Further studies dedicated to the mechanism of the cardioprotective effects of gold nanoparticles for delivering drugs and testing on different animal heart failure models, especially with relation to the age of the animal, are required. Molecular mechanisms are still not clear, and further studies are required. Different approaches for drug delivery may be suggested and should be tested, based on combination of expressions by different physical properties, e.g. sonoporation or colloid conjugation, liposomes, etc.

Study on interactions with other nanomaterials (e.g. cerium dioxide, carbon nanomaterials) and combination with other biological (gene, regenerative) therapies are recommended. After approval, agent safety development medications with future clinical testing should be initiated to implement theranostic approach for routine practice.

With the concluding points, we can formulate the following proposals (expert recommendations):

1. For the European Union (EU): Create an international project to study gold nanoparticles for the development of nanoconstructions to treat patients with heart failure. Extend studies to nanoparticle application in neurodegenerative, heart, liver and kidney diseases and muscle dystrophy, combining with biological therapies to achieve sustainable effects from theranostic approach.

2. For Ukraine: Participate in project with partners in EU to follow up experimental and clinical trials and involve related institutions and centres to the study.

## Conclusions

The review demonstrates the wide potential of nanoscale gold particles for biomedical applications because of their unique biological properties. The use of gold nanoparticles in cardiology is promising to develop fundamentally new methods of diagnosis and treatment.

## Abbreviations

AuNPs: Gold nanoparticles; AuNPs-Simdax: Conjugate of AuNPs and cardiotropic agent Simdax; BBB: Blood–brain barrier; CAs: Contrast agents; CNP: Cerium dioxide nanoparticles; GNRs: Gold nanorods; NPs: Nanoparticles; PPPM: Predictive preventive and personalised medicine; VEGF: Vascular endothelial growth factor.

## Competing interests

The authors declare that they have no competing interests.

## Authors’ contributions

MYS conceived of and organised the study and participated in the analysis. RVB performed the literature search and analysis and prepared the article. IMY, LML, NOT and ZRU participated in the data analysis. All authors read and approved the final manuscript.

## Authors’ information

MYS, Ph.D., D.Sci., Professor, is a corresponding member of the National Academy of Sciences of Ukraine and the director of the Interferon Department of Zabolotny Institute of Microbiology and Virology, NAS of Ukraine, Kyiv, Ukraine. RVB, M.D., Ph.D., is a medical doctor in the Clinical Hospital ‘Pheophania’ of State Affairs Department, National Representative of the European Association for Predictive, Preventive and Personalised Medicine (EPMA) in Ukraine. IMY, M.D., D.Sci., Professor, is the director of the Scientific-Practical Centre of Pediatric Cardiology and Cardiac Health of Ukraine, Kyiv, Ukraine, and was a Minister of Health of Ukraine (2010–2011). LLM, Ph.D., D.Sci., Professor, and TNO, Ph.D., a researcher, are members of the Interferon Department of Zabolotny Institute of Microbiology and Virology, National Academy of Sciences of Ukraine group. UZR, Ph.D., D.Sci., Professor, is the director of the Ovcharenko Institute of Biocolloidal Chemistry, National Academy of Sciences of Ukraine, Kyiv, Ukraine.

## References

[B1] Juenger J Schellberg D Kraemer S Haunstetter A Zugck C Herzog W Haass M Health related quality of life in patients with congestive heart failure: comparison with other chronic diseases and relation to functional variables Heart 2002 87 3 235 241 10.1136/heart.87.3.235 11847161 PMC1767036

[B2] Hobbs FD Kenkre JE Roalfe AK Davis RC Hare R Davies MK Impact of heart failure and left ventricular systolic dysfunction on quality of life: a cross-sectional study comparing common chronic cardiac and medical disorders and a representative adult population Eur Heart J 2002 23 23 1867 1876 10.1053/euhj.2002.3255 12445536

[B3] Neubauer S The failing heart — an engine out of fuel N Engl J Med 2007 356 11 1140 1151 10.1056/NEJMra063052 17360992

[B4] Krumholz HM Chen YT Wang Y Vaccarino V Radford MJ Horwitz RI Predictors of readmission among elderly survivors of admission with heart failure Am Heart J 2000 139 1 Pt 1 72 77 10618565 10.1016/s0002-8703(00)90311-9

[B5] Lanza GM Winter PM Caruthers SD Hughes MS Cyrus T Marsh JN Neubauer AM Partlow KC Wickline SA Nanomedicine opportunities for cardiovascular disease with perfluorocarbon nanoparticles Nanomedicine 2006 1 321 329 10.2217/17435889.1.3.321 17716162

[B6] Golubnitschaja O Costigliola V EPMA General report & recommendations in predictive, preventive and personalised medicine 2012: white paper of the European association for predictive, preventive and personalised medicine EPMA J 2012 3 14 10.1186/1878-5085-3-14 23116135 PMC3485619

[B7] Jain KK Nanomedicine: application of nanobiotechnology in medical practice Med Princ Pract 2008 17 2 89 101 10.1159/000112961 18287791

[B8] Drucker E Krapfenbauer K Pitfalls and limitations in translation from biomarker discovery to clinical utility in predictive and personalised medicine EPMA J 2013 4 7 10.1186/1878-5085-4-7 23442211 PMC3599714

[B9] Feuerstein GZ Dormer C Jr Rufollo RR Stiles G Walsh FS Rutkowski JL Translational medicine perspectives of biomarkers in drug discovery and development. Part I. Target selection and validation - biomarkers take center stage Int Drug Discovery 2007 2 5 36 43

[B10] Coelho J Ferreira P Alves P Cordeiro R Fonseca A Góis J Gil M Drug delivery systems: advanced technologies potentially applicable in personalized treatments EPMA J 2010 1 164 209 10.1007/s13167-010-0001-x 23199049 PMC3405312

[B11] Boisselier E Astruc D Gold nanoparticles in nanomedicine: preparations, imaging, diagnostics, therapies and toxicity Chem Soc Rev 2009 38 1759 1782 10.1039/b806051g 19587967

[B12] Tsai CY Shiau AL Cheng PC Shieh DB Chen DH Chou CH Yeh CS Wu CL A biological strategy for fabrication of Au/EGFP nanoparticle conjugates retaining bioactivity Nano Lett 2004 4 1209 1212 10.1021/nl049523l

[B13] Link S El-Sayed MA Spectral properties and relaxation dynamics of surface plasmon electronic oscillations in gold and silver nanodots and nanorods J Phys Chem B 1999 103 40 8410 8426 10.1021/jp9917648

[B14] Link S El-Sayed MA Shape and size dependence of radiative, non-radiative and photothermal properties of gold nanocrystals Int Rev Phys Chem 2000 19 3 409 453 10.1080/01442350050034180

[B15] Link S El-Sayed MA Optical properties and ultrafast dynamics of metallic nanocrystals Annu Rev Phys Chem 2003 54 331 366 10.1146/annurev.physchem.54.011002.103759 12626731

[B16] Huang X El-Sayed MA Gold nanoparticles optical properties and implementations in cancer diagnosis and photothermal therapy J Adv Res 2010 1 13 28 10.1016/j.jare.2010.02.002

[B17] Porter LA Ji D Westcott SL Graupe M Czernuszewicz RS Halas NJ Lee TR Gold and silver nanoparticles functionalized by the adsorption of dialkyl disulfides Langmuir 1998 14 7378 7386 10.1021/la980870i 31416111

[B18] Thompson DT Using gold nanoparticles for catalysis Nano Today 2007 2 40 43

[B19] Sperling RA Gil PR Zhang F Zanella M Biological applications of gold nanoparticles Chem Soc Rev 2008 37 1896 1908 10.1039/b712170a 18762838

[B20] Faraji AH Wipf P Nanoparticles in cellular drug delivery Bioorg Med Chem 2009 17 8 2950 2962 10.1016/j.bmc.2009.02.043 19299149

[B21] Faraday M Experimental relations of gold (and other metals) to light Philos Trans R Soc London 1857 147 145 10.1098/rstl.1857.0011

[B22] Brust M Walker M Bethell D Schiffrin DJ Whyman JR Synthesis of thiol derivatised gold nanoparticlesin a two phase liquid/liquid system Chem Soc Chem Commun 1994 7 801 802

[B23] Higashi N Kawahara J Niwa M Preparation of helical peptide monolayer-coated gold nanoparticles J. Colloid Interface Sci 2005 288 1 83 87 10.1016/j.jcis.2005.02.086 15927565

[B24] Vallhov H Qin J Johansson SM Ahlborg N Muhammed MA Scheynius A Gabrielsson S The importance of an endotoxin-free environment during the production of nanoparticles used in medical application Nano Lett 2006 6 8 1682 1686 10.1021/nl060860z 16895356

[B25] Moolhuizen G Paciotti GF Leede LG Tamarkin L Colloidal gold nanoparticles Business Briefing 2004 London: Pharmatech

[B26] Moghimi SM Hunter AC Murray JC Nanomedicine: current status and future prospects FASEB J 2005 19 311 330 10.1096/fj.04-2747rev 15746175

[B27] Praetorius NP Mandal TK Engineered nanoparticles in cancer therapy Recent Pat Drug Deliv Formul 2007 1 37 51 10.2174/187221107779814104 19075873

[B28] Elghanian R Storhoff JJ Mucic RC Letsinger RL Mirkin CA Selective colorimetric detection of polynucleotides based on the distance-dependent optical properties of gold nanoparticles Science 1997 277 1078 1081 10.1126/science.277.5329.1078 9262471

[B29] Hawley AE Davis SS Illum L Targeting of colloids to lymph nodes: influence of lymphatic physiology and colloidal characteristics Adv Drug Deliv Rev 1995 17 129 148 10.1016/0169-409X(95)00045-9

[B30] Asada K Takahashi M Kyle DJ, Osmond CB, Arntzen CJ Production and scavenging of active oxygen in photosynthesis Photoinhibition 1987 Amsterdam: Elsevier 227 287

[B31] Barath Mani Kanth S Kalishwaralal K Sriram M Pandian SRK Youn H-S Eom S Gurunathan S Anti-oxidant effect of gold nanoparticles restrains hyperglycemic conditions in diabetic mice J Nanobiotechnol 2010 8 16 10.1186/1477-3155-8-16 PMC2914719 20630072

[B32] Alvaro M Aprile C Corma A Ferrer B Garcia H Influence of radical initiators in gold catalysis: evidence supporting trapping of radicals derived from azobis(isobutyronitrile) by gold halides J Catal 2006 245 249 252

[B33] Navalon S Martin R Alvaro M Garcia H Gold on diamond nanoparticles as a highly efficient Fenton catalyst Angew Chem Int Ed Engl 2010 49 45 8403 8407 10.1002/anie.201003216 20878820

[B34] Hirst SM Karakoti A Singh S Self W Tyler R Seal S Reilly CM Bio-distribution and in vivo antioxidant effects of cerium oxide nanoparticles in mice Environ Toxicol 2013 28 2 107 118 10.1002/tox.20704 21618676

[B35] Hirst SM Karakoti AS Tyler RD Sriranganathan N Seal S Reilly CM Anti-inflammatory properties of cerium oxide nanoparticles Small 2009 5 24 2848 2856 10.1002/smll.200901048 19802857

[B36] Colon J Hsieh N Ferguson A Kupelian P Seal S Jenkins DW Baker CH Cerium oxide nanoparticles protect gastrointestinal epithelium from radiation-induced damage by reduction of reactive oxygen species and upregulation of superoxide dismutase 2 Nanomedicine 2010 6 698 10.1016/j.nano.2010.01.010 20172051

[B37] Wason MS Zhao J Cerium oxide nanoparticles: potential applications for cancer and other diseases Am J Transl Res 2013 5 2 126 131 23573358 PMC3612509

[B38] Alili L Sack M Karakoti AS Teuber S Puschmann K Hirst SM Reilly CM Zanger K Stahl W Das S Seal S Brenneisen P Combined cytotoxic and anti-invasive properties of redox-active nanoparticles in tumor-stroma interactions Biomaterials 2011 32 2918 2929 10.1016/j.biomaterials.2010.12.056 21269688

[B39] Alili L Sack M von Montfort C Giri S Das S Carroll K Zanger K Seal S Brenneisen P Downregulation of tumor growth and invasion by redox-active nanoparticles Antioxid Redox Signal 2013 10.1089/ars.2012.4831 PMC3752511 23198807

[B40] Babenko LP Zholobak NM Shcherbakov AB Voychuk SI Lazarenko LM Spivak MY Antibacterial activity of cerium colloids against opportunistic microorganisms in vitro Mikrobiol Z 2012 74 3 54 62 22830198

[B41] Zholobak NM Olevinskaia ZM Spivak NI Shcherbakov AB Ivanov VK Usatenko AV Antiviral effect of cerium dioxide nanoparticles stabilized by low-molecular polyacrylic acid Mikrobiol Z 2010 72 3 42 47 Russian 20695228

[B42] Menchón C Martín R Apostolova N Victor VM Alvaro M Herance JR García H Gold nanoparticles supported on nanoparticulate ceria as a powerful agent against intracellular oxidative stress Small 2012 8 12 1895 1903 10.1002/smll.201102255 22454217

[B43] Tsai CY Shiau AL Chen SY Chen YH Cheng PC Chang MY Chen DH Chou CH Wang CR Wu CL Amelioration of collagen-induced arthritis in rats by nanogold Arthritis Rheum 2007 56 2 544 554 10.1002/art.22401 17265489

[B44] Selvakannan PR Mandal S Phadtare S Pasricha R Sastry M Capping of gold nanoparticles by the amino acid lysine renders them water-dispersible Langmuir 2003 19 3545 3549 10.1021/la026906v

[B45] Mukherjee P Bhattacharya R Wang P Wang L Basu S Nagy JA Atala A Mukhopadhyay D Soker S Antiangiogenic properties of gold nanoparticles Clin Cancer Res 2005 11 3530 3534 10.1158/1078-0432.CCR-04-2482 15867256

[B46] Bhattacharya R Mukherjee P Xiong Z Atala A Soker S Mukhopadhyay D Gold nanoparticles inhibit VEGF165-induced proliferation of HUVEC cells Nano Lett 2004 4 2479 2481 10.1021/nl0483789

[B47] Haes AJ Hall WP Chang L Klein WL Van Duyne RP A localized surface plasmon resonance biosensor: first steps toward an Alzheimer's disease Assa Nano Lett 2004 4 1029 1034 10.1021/nl049670j

[B48] Haes AJ Chang L Klein WL Van Duyne RP Detection of a biomarker for Alzheimer's disease from synthetic and clinical samples using a nanoscale optical biosensor J Am Chem Soc 2005 127 2264 2271 10.1021/ja044087q 15713105

[B49] Bowman M-C Ballard TE Ackerson CJ Feldheim DL Margolis DM Melander C Inhibition of HIV fusion with multivalent gold nanoparticles J Am Chem Soc 2008 130 6896 6897 10.1021/ja710321g 18473457 PMC2916654

[B50] Xi D Luo X Ning Q Lu Q Yao K Liu Z The detection of HBV DNA with gold nanoparticle gene probes J Nanjing Med Univ 2007 21 4 207 212 10.1016/S1007-4376(07)60047-1

[B51] Baptista PV Koziol-Montewka M Paluch-Oles J Doria G Franco R Gold-nanoparticle-probe–based assay for rapid and direct detection of *Mycobacterium tuberculosis* DNA in clinical samples Clin Chem 2006 52 1433 1434 10.1373/clinchem.2005.065391 16798971

[B52] Baptista P Pereira E Eaton P Doria G Miranda A Gomes I Quaresma P Franco R Gold nanoparticles for the development of clinical diagnosis methods Anal Bioanal Chem 2008 391 3 943 950 10.1007/s00216-007-1768-z 18157524

[B53] Paleos CM Tsiourvas D Sideratou Z Tziveleka L Acid- and salt-triggered multifunctional poly(propylene imine) dendrimer as a prospective drug delivery system Biomacromolecules 2004 5 524 529 10.1021/bm030068h 15003016

[B54] Kisak ET Coldren B Evans CA Boyer C Zasadzinski JA The vesosome — a multicompartment drug delivery vehicle Curr Med Chem 2004 11 199 219 10.2174/0929867043456197 14754417

[B55] Wu W Wieckowski S Pastorin G Benincasa M Klumpp C Briand JP Gennaro R Prato M Bianco A Targeted delivery of amphotericin B to cells by using functionalized carbon nanotubes Angew Chem Int Ed 2005 44 6358 6362 10.1002/anie.200501613 16138384

[B56] Salem AK Searson PC Leong KW Multifunctional nanorods for gene delivery Nat Mater 2003 2 668 671 10.1038/nmat974 12970757

[B57] Brown SD Nativo P Smith J-A Stirling D Edwards PR Venugopal B Flint DJ Plumb JA Graham D Wheate NJ Gold nanoparticles for the improved anticancer drug delivery of the active component of oxaliplatin J Am Chem Soc 2010 132 4678 4684 10.1021/ja908117a 20225865 PMC3662397

[B58] Ghosh P Han G De M Kim CH Rotello VM Gold nanoparticles in delivery applications Adv Drug Delivery Rev 2008 60 1307 1315 10.1016/j.addr.2008.03.016 18555555

[B59] Edlund U Albertsson AC Polyesters based on diacid monomers Adv Drug Deliv 2003 55 585 609 10.1016/S0169-409X(03)00036-X 12706051

[B60] Lim SB Ruminstein I Onyuksel H Freeze drying of peptide drugs self-associated with long-circulating, biocompatible and biodegradable sterically stabilized phospholipid nanomicelles Int J Pharm 2008 356 345 350 10.1016/j.ijpharm.2008.01.014 18289811 PMC2413132

[B61] Ebessen M Jensen TJ Nanomedicine: techniques, potentials, and ethical implications J Biomed Biotechnol 2006 1 1 11 10.1002/biot.200690016 PMC1779503 17489016

[B62] Checkman IS Nanopharmacology 2011 Kyiv: Zadruha 424

[B63] Yu-Hsin W Ai-Ho L Jia-Yu L Cheng-Ru L Cheng-Ham W Tzu-Min L Churng-Ren W Pai-Chi L Enhanced delivery of gold nanoparticles by acoustic cavitation for photoacoustic imaging and photothermal therapy SPIE Proceedings 8581, Photons Plus Ultrasound: Imaging and Sensing: 2013 February 2; San Francisco 2013 858123 10.1117/12.2005870

[B64] Mittler R Oxidative stress, antioxidants and stress tolerance Trends Plant Sci 2002 7 9 405 410 10.1016/S1360-1385(02)02312-9 12234732

[B65] Sonavane G Tomoda K Sano A Ohshima H Terada H Makino KB In vitro permeation of gold nanoparticles through rat skin and rat intestine: effect of particle size Colloids Surf Biointerf 2008 65 1 1 10 10.1016/j.colsurfb.2008.02.013 18499408

[B66] Florence A The oral absorption of micro- and nanoparticulates: neither exceptional nor unusual Pharm Res 1997 14 3 259 266 10.1023/A:1012029517394 9098866

[B67] Douglas S Davies S Illum L Nanoparticles in drug delivery CRC Crit Rev Ther Drug Carrier Syst 1987 3 3 233 261 3549008

[B68] Ganeshchandra S Tomoda K Makino K Biodistribution of colloidal gold nanoparticles after intravenous administration: effect of particle size Colloids Surf Biointerf 2008 66 2 274 280 10.1016/j.colsurfb.2008.07.004 18722754

[B69] Olivier JC Drug transport to brain with targeted nanoparticles Pharmacol Rev 2005 57 173 185 10.1124/pr.57.2.4 15717062 PMC539329

[B70] Fabian G Persorption—the way of large sized corpuscle particles via the lymphatic system Lymphology 1983 16 43 48 6843178

[B71] Volkheimer G Schulz FH Lindenau A Beitz U Persorption of metallic iron particles Gut 1969 10 32 33 10.1136/gut.10.1.32 5784157 PMC1552684

[B72] Schneider HJ Dedek W Grahl R Mothes B Uhlemann J Schwarz H Schwachulla G Reuter H Mohring M Studies of the persorption of large particles from radio-labelled cation exchangers Urol Int 1983 38 116 120 10.1159/000280874 6845563

[B73] Volkheimer G Schulz FH The phenomenon of persorption Digestion 1968 1 213 218 10.1159/000196856 5696242

[B74] Toporova OK Irodov DM Bubnov RV Kholodkova OL Gulko TP Ruban TP Morgunov PV Kordium VA Intrahepatic ultrasound-mediated gene delivery J Hepatol 2013 58 S119 10.1016/S0168-8278(13)60283-4

[B75] Eghtedari M Oraevsky A Copland JA Kotov NA Conjusteau A Motamedi M High sensitivity of in vivo detection of gold nanorods using a laser optoacoustic imaging system Nano Lett 2007 7 7 1914 1918 10.1021/nl070557d 17570730

[B76] Conversano F Soloperto G Greco A Ragusa A Casciaro E Chiriacò F Demitri C Gigli G Maffezzoli A Casciaro S Echographic detectability of optoacoustic signals from low-concentration PEG-coated gold nanorods Int J Nanomedicine 2012 7 4373 4389 22927756 10.2147/IJN.S33908 PMC3420597

[B77] Pan D Pramanik M Senpan A Ghosh S Wickline SA Wang LV Lanza GM Near infrared photoacoustic detection of sentinel lymph nodes with gold nanobeacons Biomaterials 2010 31 4088 4093 10.1016/j.biomaterials.2010.01.136 20172607 PMC2874457

[B78] Jain PK El-Sayed IH El-Sayed MA Au nanoparticles target cancer Nano Today 2007 2 18 29

[B79] Pan D Pramanik M Senpan A Wickline SA Wang LV Lanza GM A facile synthesis of novel self-assembled gold nanorods designed for near-infrared imaging J Nanosci Nanotechnol 2010 10 8118 8123 10.1166/jnn.2010.3034 21121304 PMC3096062

[B80] Song KH Kim C Maslov K Wang LV Noninvasive in vivo spectroscopic nanorod-contrast photoacoustic mapping of sentinel lymph nodes Eur J Rad 2009 70 227 231 10.1016/j.ejrad.2009.01.045 PMC7015035 19269762

[B81] Mallidi S Larson T Tam J Joshi PP Karpiouk A Sokolov K Emelianov S Multiwavelength photoacoustic imaging and plasmon resonance coupling of gold nanoparticles for selective detection of cancer Nano Lett 2009 9 8 2825 2831 10.1021/nl802929u 19572747 PMC2898720

[B82] Lu W Huang Q Ku G Wen X Zhou M Guzatov D Brecht P Su R Oraevsky A Wang LV Li C Photoacoustic imaging of living mouse brain vasculature using hollow gold nanospheres Biomaterials 2010 31 9 2617 2626 10.1016/j.biomaterials.2009.12.007 20036000 PMC2813997

[B83] Li ML Wang JC Schwartz JA Gill-Sharp KL Stoica G Wang LV In-vivo photoacoustic microscopy of nanoshell extravasation from solid tumor vasculature J Biomed Opt 2009 14 1 010507 10.1117/1.3081556 19256687 PMC6988903

[B84] Gibson JD Khanal BP Zubarev ER Paclitaxel-functionalized gold nanoparticles J Am Chem Soc 2007 129 11653 11661 10.1021/ja075181k 17718495

[B85] Kogan MJ Bastus NG Amigo R Grillo-Bosch D Araya E Turiel A Labarta A Giralt E Puntes VF Nanoparticle-mediated local and remote manipulation of protein aggregation Nano Lett 2006 6 1 110 115 10.1021/nl0516862 16402797

[B86] Shukla R Chanda N Zambre A Upendran A Katti K Kulkarni RR Nune SK Casteel SW Smith CJ Vimal J Boote E Robertson JD Kan P Engelbrecht H Watkinson LD Carmack TL Lever JR Cutler CS Caldwell C Kannan R Katti KV Laminin receptor specific therapeutic gold nanoparticles (198AuNP-EGCg) show efficacy in treating prostate cancer Proc Natl Acad Sci USA 2012 109 31 12426 12431 10.1073/pnas.1121174109 22802668 PMC3411993

[B87] Chen J Wiley BJ Xia Y One-dimensional nanostructures of metals: large-scale synthesis and some potential applications Langmuir 2007 23 8 4120 4129 10.1021/la063193y 17249708

[B88] Kim K Kim JH Park H Kim YS Park K Nam H Lee S Park JH Park RW Kim IS Choi K Kim SY Park K Kwon IC Tumor-homing multifunctional nanoparticles for cancer theragnosis: simultaneous diagnosis, drug delivery, and therapeutic monitoring J Control Release 2010 146 2 219 227 10.1016/j.jconrel.2010.04.004 20403397

[B89] Stuchinskaya T Moreno M Cook MJ Edwards DR Russell DA Targeted photodynamic therapy of breast cancer cells usingantibody–phthalocyanine–gold nanoparticle conjugates Photochem Photobiol Sci 2011 10 822 831 10.1039/c1pp05014a 21455532

[B90] Larguinho M Baptista PV Gold and silver nanoparticles for clinical diagnostics - from genomics to proteomics J Proteomics 2012 75 10 2811 2823 10.1016/j.jprot.2011.11.007 22119545

[B91] Cheng C Chang K-C Chen C-S Pijanowska DG Biosensor with nano-gold particle modified pencil lead carbon electrode for long-term glucose monitoring of waste tree branch hydrolysis J Chin Chem Soc 2011 58 6 739 748 10.1002/jccs.201190116

[B92] Perrault SD Chan WCW In vivo assembly of nanoparticle components to improve targeted cancer imaging Proc Nat Acad Sci USA 2010 107 11194 11199 10.1073/pnas.1001367107 20534561 PMC2895069

[B93] Nam JM Thaxton CS Mirkin CA Nanoparticle-based bio-bar codes for the ultrasensitive detection of proteins Science 2003 301 1884 1886 10.1126/science.1088755 14512622

[B94] Godin B Sakamoto JH Serda RE Grattoni A Bouamrani A Ferrari M Emerging applications of nanomedicine for the diagnosis and treatment of cardiovascular diseases Trends Pharmacol Sci 2010 31 5 199 205 10.1016/j.tips.2010.01.003 20172613 PMC2862836

[B95] Kong DF Goldschmidt-Clermont PJ Tiny solutions for giant cardiac problems Trends Cardiovasc Med 2005 15 6 207 211 10.1016/j.tcm.2005.07.003 16182130

[B96] Chnari E Nikitczuk JS Uhrich KE Moghe PV Nanoscale anionic macromolecules can inhibit cellular uptake of differentially oxidized LDL Biomacromolecules 2006 7 597 603 10.1021/bm0506905 16471936

[B97] Hsieh PC Davis ME Gannon J MacGillivray C Lee RT Controlled delivery of PDGFBB for myocardial protection using injectable self-assembling peptide nanofibers J Clin Invest 2006 116 237 248 16357943 10.1172/JCI25878 PMC1312017

[B98] Davis ME Hsieh PC Takahashi T Song Q Zhang S Kamm RD Grodzinsky AJ Anversa P Lee RT Local myocardial insulin-like growth factor 1 (IGF-1) delivery with biotinylated peptide nanofibers improves cell therapy for myocardial infarction Proc Natl Acad Sci USA 2006 103 8155 8160 10.1073/pnas.0602877103 16698918 PMC1472445

[B99] Sy JC Davis ME Delivering regenerative cues to the heart: cardiac drug delivery by microspheres and peptide nanofibers J Cardiovasc Transl Res 2010 3 5 461 468 10.1007/s12265-010-9210-x 20628908 PMC3024054

[B100] Powrie F Coffman RL Cytokine regulation of T-cell function: potential for therapeutic intervention Trends Pharmacol Sci 1993 14 5 164 168 10.1016/0165-6147(93)90202-U 8105593

[B101] Astry B Harberts E Moudgil KD A cytokine-centric view of the pathogenesis and treatment of autoimmune arthritis J Interferon Cytokine Res 2011 31 12 927 940 10.1089/jir.2011.0094 22149412 PMC3234492

[B102] Mill JG Stefanon I dos Santos L Baldo MP Remodeling in the ischemic heart: the stepwise progression for heart failure Braz J Med Biol Res 2011 44 9 890 898 10.1590/S0100-879X2011007500096 21829898

[B103] Bubnov RV Evidence-based pain management: is the concept of integrative medicine applicable? EPMA J 2012 3 13 10.1186/1878-5085-3-13 23088743 PMC3533862

[B104] Bubnov R Yevseenko V Semeniv I Ultrasound guided injections of platelets rich plasma for muscle injury in professional athletes. Comparative study Med Ultrason 2013 15 2 101 105 10.11152/mu.2013.2066.152.rb1vy2 23702498

[B105] Andrews RJ Neuroprotection at the nanolevel – part I: introduction to nanoneurosurgery Ann NY Acad Sci 2007 1122 169 184 10.1196/annals.1403.012 18077572

[B106] Doggrell SA Brown L Rat models of hypertension, cardiac hypertrophy and failure Cardiovasc Res 1998 39 1 89 105 10.1016/S0008-6363(98)00076-5 9764192

[B107] Kannel WB Hypertension as a risk for cardiac events – epidemiological results of long-term studies J Cardiovasc Pharmacol 1993 21 Suppl 2 S27 S37 7692148 10.1097/00005344-199321002-00006

[B108] Bubnov RV The use of ultrasound equipment of general use for in vivo study of cerium dioxide nanoparticles introduction in mice Ultrasound Med Biol 2011 37 8 S162 10.1016/j.ultrasmedbio.2011.05.750

[B109] Spivak M Bubnov R Yemets I Lazarenko L Timoshok N Vorobieva A Mohnatyy S Ulberg Z Reznichenko L Grusina T Zhovnir V Zholobak N Doxorubicin dose for congestive heart failure modeling and the use of general ultrasound equipment for evaluation in rats. Longitudinal in vivo study Med Ultrason 2013 15 1 23 28 10.11152/mu.2013.2066.151.ms1ddc2 23486620

[B110] Trikas A Antoniades C Latsios G Vasiliadou K Karamitros I Tousoulis D Tentolouris C Stefanadis C Long-term effects of levosimendan infusion on inflammatory processes and sFas in patients with severe heart failure Eur J Heart Fail 2006 8 8 804 809 10.1016/j.ejheart.2006.03.003 16713737

[B111] Seaton A Tran L Aitken R Donaldson K Nanoparticles, human health hazard and regulation J R Soc Interface 2010 7 119 129 10.1098/rsif.2009.0252.focus PMC2843982 19726441

[B112] Murphy CJ Gole AM Hunyadi SE Stone JW Sisco P Alkilany A Hankins PL Kinard B Chemical sensing and imaging with metallic nanorods Chem Comm 2008 41 1721 1730 10.1039/b711069c 18209787

[B113] Son SJ Bai X Lee SB Inorganic hollow nanoparticles and nanotubes in nanomedicine. Part 2: imaging, diagnostic, and therapeutic applications Drug Discov Today 2007 12 15–16 657 653 17706548 10.1016/j.drudis.2007.06.012

[B114] Shukla R Bansal V Chaudhary M Basu A Bhonde RR Sastry M Biocompatibility of gold nanoparticles and their endocytotic fate inside the cellular compartment: a microscopic overview Langmuir 2005 21 10644 10654 10.1021/la0513712 16262332

[B115] Duffin R Tran L Brown D Stone V Donaldson K Proinflammogenic effects of low-toxicity and metal nanoparticles in vivo and in vitro: highlighting the role of particle surface area and surface reactivity Inhal Toxicol 2007 19 10 849 856 10.1080/08958370701479323 17687716

